# Estimating the palliative effect of percutaneous endoscopic gastrostomy in an observational registry using principal stratification and generalized propensity scores

**DOI:** 10.1038/srep33431

**Published:** 2016-09-19

**Authors:** Pallavi S. Mishra-Kalyani, Brent A. Johnson, Jonathan D. Glass, Qi Long

**Affiliations:** 1 Food and Drug Administration, Office of Biostatistics, Silver Spring, 20903, USA; 2 University of Rochester, Department of Biostatistics and Computational Biology, Rochester, 14642, USA; 3 Emory University, Department of Neurology, Atlanta, 30322, USA; 4 Emory University, Department of Biostatistics and Bioinformatics, Atlanta, 30322, USA

## Abstract

Clinical disease registries offer a rich collection of valuable patient information but also pose challenges that require special care and attention in statistical analyses. The goal of this paper is to propose a statistical framework that allows for estimating the effect of surgical insertion of a percutaneous endogastrostomy (PEG) tube for patients living with amyotrophic lateral sclerosis (ALS) using data from a clinical registry. Although all ALS patients are informed about PEG, only some patients agree to the procedure which, leads to the potential for selection bias. Assessing the effect of PEG is further complicated by the aggressively fatal disease, such that time to death competes directly with both the opportunity to receive PEG and clinical outcome measurements. Our proposed methodology handles the “censoring by death” phenomenon through principal stratification and selection bias for PEG treatment through generalized propensity scores. We develop a fully Bayesian modeling approach to estimate the survivor average causal effect (SACE) of PEG on BMI, a surrogate outcome measure of nutrition and quality of life. The use of propensity score methods within the principal stratification framework demonstrates a significant and positive effect of PEG treatment, particularly when time of treatment is included in the treatment definition.

Data from clinics and disease registries can offer an opportunity to examine the effect of treatment over time in a natural setting. However, the nature of such data can pose many challenges for unbiased estimation of a treatment effect, particularly in the case of diseases with high mortality rates such that “censoring by death” is a concern. In this paper, we are interested in estimating a causal effect of treatment in a clinical registry of patients with amyotrophic lateral sclerosis (ALS), a neurodegenerative disorder with a very poor prognosis^1^,^2^. However, the data feature several unique and noteworthy characteristics that require special consideration. The most obvious difficulty is that non-random treatment assignment may lead to issues of selection bias and confounding. Secondly, the data are collected longitudinally for each individual, but with varying time elapsed between each individual’s measurements and amongst all individuals. Finally, the fatal and fast-progressing nature of ALS results in the potential censoring by death of the outcome; in other words, outcome is only observed if a patient survives beyond the date on which outcome in measured.

The use of principal stratification in the context of Rubin’s Causal Model while considering unobserved outcome data as censored by death provides a framework for analysis of the ALS registry data. The principal stratification framework is described in detail by Frangakis and Rubin^3^, and Zhang and Rubin^4^ extend this methodology for stratification when the outcome is “censored” by a post-treatment variable such as survival or graduation^4^. Zhang *et al.*^5^ further outlines specific parametric approaches for identification of survivor average causal effect (SACE) in the analysis of truncation by death using principal stratification^5^. The unbiased estimation of a principal effect, defined as the causal effect within principal strata, relies on an assumption of ignorablility or no unmeasured confounding^3^. However, when selection bias or confounding may be present, either as residual confounding in a randomized clinical trial or due to observational data, Shwartz *et al.*^6^ show that the resulting principal effect estimate is likely to be biased^6^. This result indicates that in the absence of randomization or when the randomization scheme results in poor balance among treatment groups, there is a need to incorporate methods for alleviating selection bias and confounding within a principal stratification framework.

There are many methods that address selection bias or confounding in observational studies, including the propensity score introduced by Rosenbaum and Rubin^7^, which provides a means of balancing covariates across treatment groups thereby mimicking a randomized study design. Though propensity scores were introduced for balancing treatment assignment groups when treatment is binary, other authors have extended these methods to non-binary treatment assignment models, such as generalized propensity score methods^8^,^9^. Specifically, these methods allow for the estimation of causal effects when treatment assignment is ordinal, categorical, continuous, semi-continuous, or even multivariate. In the proposed methodology, the generalized propensity score is estimated using a proportional-hazards model for time to treatment.

In the following sections, we propose a framework that allows for the causal effect of treatment in the ALS registry data by combining principal stratification with adjustment of covariates using generalized propensity score. Although Jo and Stuart^10^ included propensity scores within a principal stratification framework, the scores were utilized not for removal of selection bias or confounding in the randomized data of interest, but rather for prediction of principal strata membership for a matched analysis^10^. Furthermore, propensity score methods for non-binary treatment assignment have not yet been employed for conditional ignorability in a principal stratification framework. The methodology presented in this paper addresses the practical application of the principal stratification framework to the analysis of the ALS registry data. The proposed methodology for analysis is described in detail in the Methodology section, followed by the results of the application to the data from the Emory ALS Clinic registry.

The data in the ALS registry are measured longitudinally at patient clinic visits occurring at uneven intervals of time for each individual, *i* = 1, 2, …, *N*. The baseline visit is defined as the first clinic visit for all patients, and is denoted by *t*_0_. The set of patient characteristics, denoted by **D**, includes both a set of characteristics measured only at baseline and a set that are measured at each clinic visit. Of particular interest in this data is the effect of a surgical insertion of a percutaneous endoscopic gastrostomy (PEG), a palliative procedure that provides enteral nutrition, on the outcome BMI, a proxy measure of adiposity associated with nutritional status and mortality. Denoted as **Y**, the observed outcome is the measurement of BMI collected at the clinic visit that is closest to but not past 1 year post-baseline (*t*^*o*^). Time of survival, denoted by *T*_*S*_, is measured as days from baseline until death and is used to create an indicator of survival *S* within the first year post-initial clinic visit. For those individuals who receive PEG prior to 1 year post-baseline, treatment is recorded as both the time from PEG surgery until 1 year post baseline, **T**_**Z**_, as well as a binary indicator of PEG surgery within the first year post-baseline, **Z**.

## Methodology

For data such as the Emory ALS Clinic registry, where treatment assignment can be measured as both a binary indicator of treatment and as time of treatment, different models may be postulated for the effect of treatment on outcome varying by the treatment variables used. In particular, in the proposed methodology, a dichotomous treatment model and a time of treatment model are considered. The dichotomous treatment model considers the binary definition of PEG treatment only, while the time of treatment model incorporates both the dichotomous definition of treatment as well as a measure of time elapsed from treatment until the time of outcome measurement (*t*^*o*^ − *T*_*Z*_, *i*). In each of these models, the definition of treatment assignment not only effects the interpretation of the estimated treatment effect but also the modeling and calculation of the propensity score. The final estimates of treatment effect as determined by the causal inference framework outlined the the subsequent sections results are causal effect estimates. Specifically, we estimate an effect of the decision to recieve treatment at any time point and the effect of delaying treatment post-baseline

Patient characteristics in combination with time to treatment, indicator of no treatment, and the propensity score vector **PS**, as described in the following section, comprise the matrix of observed data **X**, which is used in parts for modeling. In the stratified regression model for the outcome **Y**, the subset of **X** that is included in the analysis is **X**_1_, which may include **T**_**Z**_, **Z**, **PS**, and a subset of variables from **D**, depending on the outcome model and the stratum. **X**_2_ is the subset of **X** included in the regression model for the principal strata probabilities *P*(*G* = *g*), which may include **PS** and a subset of variables from **D**.

### Framework for Causal Inference

Two commonly used assumptions are made in this causal inference framework. First, we make the Stable Unit Treatment Value Assumption (SUTVA) as defined by Cox in 1958 and summarized by Rubin^11^. This assumption states that there is no interference among the potential outcomes of one individual and the treatment choices of another individual. Secondly, we assume Strong Ignorability of Treatment Assignment, which states the distribution of the potential outcomes is independent of treatment assignment, given the observed covariates^7^. The ignorability assumption often proves to be non-trivial, particularly for data from observational studies.

As earlier defined, the indicator for treatment from baseline until *t*^*o*^ (time of outcome measurement) is **Z**, the outcome of interest is **Y**, and the post-treatment variable of survival is **S**. Using the Rubin Causal Model as a framework for causal inference, we can define potential outcomes 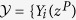, 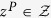 for 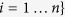 and 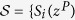, 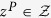 for 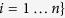, where 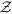 is the set of potential treatment values and *Y*_*i*_(*z*^*P*^) and *S*_*i*_(*z*^*P*^) are the potential outcomes for a given potential treatment *z*^*P*^^12^.

### Propensity Scores and Generalized Propensity Scores

When considering only the effect of a dichotomous treatment, standard propensity scores may be employed. The individual propensity scores are estimated using a logistic regression model for the probability of treatment using all available baseline patient characteristics, **D**. Specifically, we model *logit (P (Z* = 1)) = **D**^*T*^ *β*, and use the parameter estimate 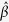 to calculate the individual probabilities *P (Z*_*i*_ = 1) as each individual’s propensity score, *PS*_*i*_.

However, when considering a model for the outcome that identifies associations with time of treatment, we must consider a more flexible model for propensity score. The generalized propensity score methods proposed by Imai and van Dyk^8^ and Hirano and Imbens^9^ allow the inclusion of the information provided by covariates and more importantly control for selection bias and confounding when a non-binary treatment assignment model is considered in a non-randomized sample^8^,^9^,^13^. It is noteworthy that these two generalized propensity score methods are equivalent when estimating propensity scores at fixed time points, even if time-dependent models are used.

Both Imai and Van Dyk and Hirano and Imbens derive “large-sample” theoretical results of balancing properties and ignorable treatment assignment that resemble the results for the standard propensity score proposed by Rosenbaum and Rubin^7^. Specifically, Imai and Van Dyk^8^ show that given the assumptions of SUTVA, Strong Ignorability of Treatment Assignment, and a uniquely parameterized propensity function (which allows for the propensity function to be represented by this unique parameter *θ* which also characterizes the relationship with **D**), the propensity function is a balancing score. The balancing property can be assessed by regressing each covariate on treatment assignment with adjustment for estimated propensity function (by matching or stratification). The balancing property allows for a result of strong ignorability of treatment assignment given the propensity function. Therefore, using the aforementioned assumptions and results, for time to treatment we may use a Cox proportional hazards model *h*(*t*) = *h*_0_(*t*) exp(**D**^*T*^ *β*), and the estimated linear predictor 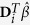 is the propensity function or generalized propensity score, thereby removing any confounding by the time to treatment assignment.

The estimated propensity scores, as defined for dichotomous treatment model and for the time of treatment model, are included in both the model for the outcome (**Y**) as well as the model determining principal strata (**G**), to control for issues of selection bias and confounding. It is noteworthy that inclusion of the propensity scores in the principal strata model is necessary in the absence of randomization, as otherwise the principal effect is likely to be biased. To allow flexibility in the control for selection bias when including each of the propensity scores a linear predictor, the use of quadratic and cubic polynomial higher order terms are also considered for each propensity score.

### Principal Stratification

If a patient is not alive under the treatment that is actually received, *S*_*i*_ = 0, the outcome *Y*_*i*_ cannot be measured. We may consider those outcomes that are not measured due to patient death as not defined on the set of real positive numbers, **R**^+^. Following the notation of Zhang and Rubin^4^, we can instead consider the non-observed outcomes to be *, extending our sample space to 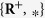. In the presence of this censoring of the outcome by death, principal stratification using post treatment survival status allows for estimation of the treatment effect. Specifically, the Survivor Average Causal Effect (SACE) is defined as the mean difference in the outcomes of treated individuals compared to untreated individuals in the *LL* stratum, *E*(*Y*_*LL*,*i*_(1)) − *E*(*Y*_*LL*,*i*_(0)). The four potential principal strata are constructed by pairing indicators of survival by treatment scenarios at time of outcome measurement, as defined below.

- *LL* = {*i*|*S*_*i*_(1) = 1, *S*_*i*_(0) = 1}, or those patients who would be alive at time *t*^*o*^ regardless of treatment.
- *LD* = {*i*|*S*_*i*_(1) = 1, *S*_*i*_(0) = 0}, or those patients who at time *t*^*o*^ would be alive if they receive the PEG tube but would not be alive if they did not.
- *DL* = {*i*|*S*_*i*_(1) = 0, *S*_*i*_(0) = 1}, or those patients who at time *t*^*o*^ would not be alive if they receive PEG treatment, but would be alive if they did not.
- *DD* = {*i*|*S*_*i*_(1) = 0, *S*_*i*_(0) = 0}, or those patients who would not be alive at time *t*^*o*^ regardless of treatment.

The probabilities of the four strata (*π*_*LL*_, *π*_*LD*_, *π*_*DL*_, and *π*_*DD*_) can be modeled with a multinomial logit model using a subset of the observed covariate matrix **X**, **X**_**2**_, which must include **PS** and may include patient characteristics **D**. The probability of an individual being in principal strata *g* is given in equation (1). As in any multinomial logit model, one category must be selected as a reference group.


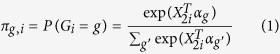


However, for any given individual *i*, we only observe the survival outcome given the observed treatment status. These four groups based on observed data are defined as follows:

- *O*(1, 1) = {*i*|*Z*_*i*_ = 1, 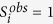}: individuals who are treated and are alive at time *t*^*o*^
- *O*(1, 0) = {*i*|*Z*_*i*_ = 1, 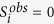}: individuals who are treated and are not alive at time *t*^*o*^
- *O*(0, 1) = {*i*|*Z*_*i*_ = 0, 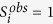}: individuals who are not treated and are alive at time *t*^*o*^
- *O*(0, 0) = {*i*|*Z*_*i*_ = 0, 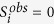}: individuals who are not treated and are not alive at time *t*^*o*^

These observed groups are composed of mixtures of the principal strata. In other words, *O*(1, 1) is comprised of a mixture of individuals from the *LL* and *LD* strata, *O*(1, 0) is comprised of individuals from the *LL* and *DL* strata, *O*(0, 1) is comprised of individuals from the *DD* and *DL* strata, and *O*(0, 0) is comprised of individuals from the *DL* and *DD* strata.

Thus far, the framework for principal stratification does not employ an assumption of monotonicity. This assumption implies that the DL stratum (those who do not live with the receipt of treatment, but will live if untreated) does not exist. Other than the reduction of principal strata to three rather than four, the model framework is largely the same under the monotonicity assumption. For completeness of methodology and to test the sensitivity of the results to this assumption, results for all data analysis and simulation studies are reported with and without the monotonicity assumption.

### Bayesian Framework for Estimation and Inference

The observed outcome *Y*_*i*_, which is only observed when *S*_*i*_ = 1, is assumed to have a normal distribution, *f*_*g*_, within each of the principal strata and with parameters and covariates that differ by strata. Specifically, the outcome distributions are defined as 
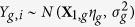
 for *g* ∈ *LL*, *LD*, *DL*. **X**_1,*LL*_ includes the column for intercept, one or both of the treatment variables depending on the treatment assignment model considered, and the estimated propensity score corresponding ot the treatment assignment model. The outcome models for the *LD* and *DL* strata do not include any treatment covariates as the individuals with an observed outcome in each of these strata are either all treated or all untreated, respectively. Therefore, *X*_1,*LD*_ and *X*_1,*DL*_ include columns for intercept and propensity score only.

Using the stratified distributions and the probability of each principal stratum, the structure of the observed data likelihood for any individual and for all possible combinations of *Z*_*i*_ and *S*_*i*_ is given in Table 1. Each cell value is the likelihood of the observed data if the values of the individuals’ strata are known. Thus the conditional probability of *G*_*i*_ = *g* given the observed data is the ratio of each cell to the total of that row. Rows *O*(1, 0) and *O*(0, 0) are included in this table for a comprehensive understanding of the possible combinations of treatment and survival, but individuals who fall into these groups do not have outcome data that will contribute to the observed data likelihood since *S*_*i*_ = 0 and thus *Y*_*i*_ is unobserved. Therefore, individuals in this group will only contribute to the model for the probability of principal strata, which is reflected in the observed data likelihood in the Appendix I.

Prior distributions for the specified parameters in the observed data likelihood should be chosen carefully, with attention to distributions that may be informative, proper, and conjugate where appropriate. For this analysis, conjugate multivariate normal and inverse-gamma distributions are assigned for the prior distributions of the different forms of *η*_*g*_ and 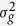 respectively. The prior distributions for *α*_*g*_ are non-informative and are proportional to 1. Details of each prior distribution are provided in Appendix II.


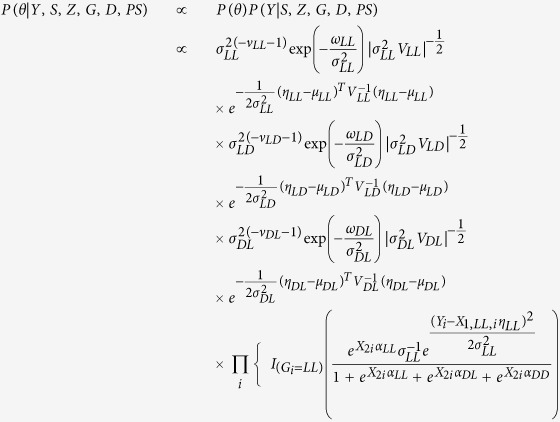



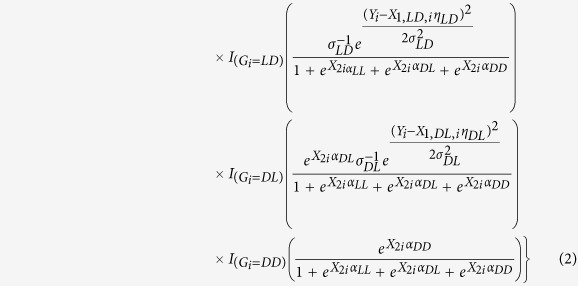


The posterior distribution of the parameters given the observed data likelihood and specified prior distributions is provided in equation (2). Though the principal stratum of each individual is unknown, the observed treatment and survival groups may be used to inform imputation of the principal strata assignments. One option in Bayesian analysis is the Data Augmentation (DA) algorithm^14^, which treats *G* as missing data, imputes *G*, and subsequently simulates the posterior distributions of *θ*, a given imputed *G*.

The DA algorithm is employed by using two iterative and alternating steps to simulate a complete data likelihood and allow for posterior inference. The first step, the Imputation or I-step, imputes the value of the principal strata *G*_*i*_ for each individual. This is accomplished by using the parameter values 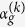, 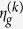, and 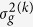 from the current approximation of posterior (from the *k*th iteration) to generate 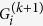 by using the conditional probabilities that are given by taking the ratio of cell value to row total in Table 1. The conditional probabilities, *ρ*_*O*,*i*_ are used in a Bernoulli distribution that imputes individual membership to one of the two principal strata that correspond with the observed group *O* (see Appendix III). More specifically, at the (*k* + 1) iteration, each individual has a probability of being in a stratum that depends on their observed values (*Z*_*i*_, *S*_*i*_, *Y*_*i*_, *PS*_*i*_).

The P-step, or Posterior step, is then employed by using the imputed complete data set, and the parameters 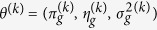 can be updated to 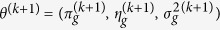 by sampling from the full conditional distributions of each parameter, or the distribution in which all other parameters in *θ* are conditioned upon. Either the Gibbs Sampler or the Metropolis-Hastings (MH) Algorithm may be employed for sampling, with choice of algorithm influenced by the type of full conditional distribution.

## Analysis of the Emory ALS Clinic Data

The ALS registry dataset includes data from 729 patients who visited the Emory ALS clinic at least once from January 1, 1997 and July 31, 2011. All individuals were diagnosed with ALS prior to first clinic visit, and none had received PEG treatment. Of these patients, 38 patients were excluded for not having any follow up clinic visits within 1 year of their first clinic visit, 25 were excluded for having extremely long survival times (>5 years post-baseline), and 86 were excluded for having no post-baseline measurements of the outcome BMI within the first year of follow up. Characteristics measured at baseline for each individual include sex, site of ALS onset, age at diagnosis, BMI at baseline, and days from diagnosis to first clinic visit (Δ*T*_*DX*_). Additionally, some individual characteristics are measured at each clinic visit including forced vital capacity (FVC), change in FVC from baseline 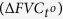, change in BMI from baseline 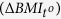, and total number of clinic visits. Those characteristics that are measured as continuous variables (namely age at diagnosis, BMI at baseline, FVC, and change in FVC from baseline) are normalized before inclusion as covariates for the propensity score model, principal strata model, or outcome model.

A comparison of those who receive treatment within one year of follow-up and those who do not among the remaining 580 individuals in the ALS registry is available in Table 2. Of the 200 treated patients 41.5% or 83 individuals are alive one year from baseline, while of the 384 untreated individuals 54.2% or 206 individuals are alive at this time-point (p < 0.01). In general, treated individuals tend to have characteristics that align with greater risk of advanced disease, such as smaller values of mean FVC, increased age, lower proportions of spinal onset, and higher proportions of females.

Baseline measurements of BMI are not significantly different among the treated and untreated populations were not significant at a level of *α* = 0.05, however, FVC measurements taken at baseline are significantly different (p < 0.01), with treated individuals having a lower mean measurement than untreated individuals. Demographic characteristics such as age at diagnosis and sex are also significantly different among treated and untreated, with treated individuals being about 3 years older and more likely to be female than untreated individuals (p < 0.01 and p = 0.02 respectively). Additionally, the proportion of patients with spinal onset of disease is significantly lower in the treated population (p < 0.01), which along with the lower mean FVC at baseline, higher age, and greater proportion of females indicates increased risk of advanced disease in the treated patient population.

When considering time of outcome measurement, 1 year post-baseline, the clinical measurements of FVC in patients who are treated and untreated have an even greater gap than the measurements at baseline (p < 0.01). This result may imply that those individuals who do receive treatment within one year post-baseline are not only in poorer condition at baseline, but they are generally in poorer condition as time and their disease progresses. BMI is also lower for patients who are treated when compared to untreated (p = 0.05).

### Balance of Covariates

To test the balance of covariates using propensity score methods, the association of patient characteristics with the indicator of treatment was examined for change after conditioning on the proposed propensity scores. Table 3 presents the p-values of these associations when examined marginally, conditioned on the standard propensity score as calculated from a logistic regression of the treatment indicator, and conditioned on the generalized propensity score as calculated from a Cox proportional hazards regression of the time to treatment.

Overall, the use of propensity scores does balance the covariates across treatment groups. Most patient characteristics have a significant marginal association with treatment. After conditioning on the standard propensity score, all associations of patient characteristics with treatment become non-significant, indicating that balance of covariates is successfully achieved. Conditioning on the generalized propensity score is also fairly successful in balancing covariates, with most patient characteristic associations with treatment becoming non-significant. Though two patient characteristics (baseline FVC and number of visits in the first year of follow-up post-baseline) remain associated with treatment after conditioning on the generalized propensity score.

## Results

SACE of PEG Treatment is estimated in 16 model scenarios; in addition to the two definitions of treatment (binary indicator of treatment and time of treatment), models are considered for various levels of propensity score inclusion (none, linear, quadratic, and cubic propensity score terms) and with or without the assumption of monotonicity. For all analyses, the MCMC algorithm was run for a total of 10,000 iterations, with a burn-in period of 5000 iterations.

Table 4 presents a comparison of models with and without the linear propensity score or generalized propensity score terms. Overall, the inclusion of a propensity score may change the magnitude, direction and, significance of the treatment effect estimates. In the case of the binary treatment indicator only model, though the treatment effect remains negative and non-significant, the magnitude of the effect of treatment on BMI is closer to zero after including propensity scores. For comparison, Table 5 includes the results of the PEG treatment effect estimates in all eight variations of the binary treatment model. When all four strata are considered, the treatment effect is similar when any propensity score terms are included in the model. The application of the monotonicity assumption does cause some minor discrepancies in the magnitude and direction of the effect estimates in the models with propensity score terms. However, all treatment effect estimates are non-significant as all credible intervals include the null value of 0. Therefore, there seems to be no significant treatment effect in the model with binary indicator of PEG treatment only.

The time of treatment model, which includes both time from treatment to 1 year post-baseline and a binary indicator of treatment, shows a change not only in the magnitude but also in the significance of the effect estimates of PEG treatment when comparing the results from inclusion of linear GPS term to that without a GPS term (Table 4). Most notably, when GPS are used, the effect of the PEG treatment indicator is larger in magnitude and is significant, compared to a smaller non-significant estimate when no GPS terms are included in the model. These results remain significant after including higher order GPS terms and employing the monotonicity assumption (Table 6).

Though the results in this model with time from treatment and binary indicator of treatment seem promising in providing a positive treatment effect of PEG, a careful interpretation of the treatment effect estimates is necessary. While there is a significant negative effect on BMI for each unit increase in months of time from treatment to one year post-baseline (−0.34), this effect is additive to the binary treatment indicator at any time. Thus, when the time from treatment to 1 year post-baseline is small, there is an overall positive effect of treatment in the measurement of BMI one year post-baseline.

**Figure 1:**
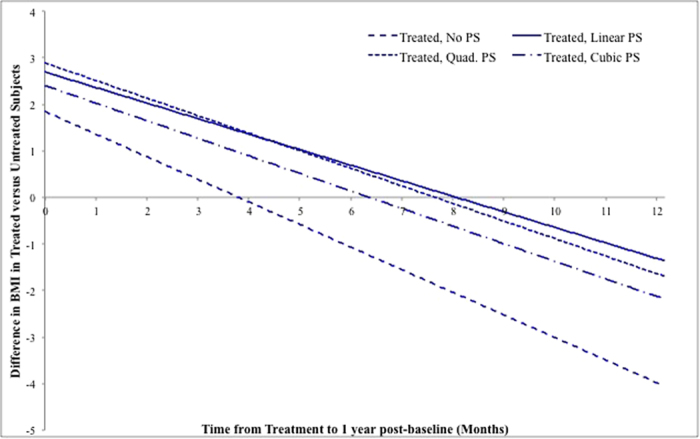
Difference in average BMI of treated compared to untreated individuals over time from treatment to 1 year post-baseline (N = 491).

To better visualize the effect of treatment in the time of treatment model, the difference in mean BMI of treated individuals and untreated individuals is plotted over time from treatment to one year post-baseline in Fig. 1. As the time between treatment and one year post-baseline increases, the effect of PEG treatment diminishes. One possibility is that this may be due to a waning effect of treatment over time; as outcome is measured further from the time of treatment, the effect of PEG treatment may no longer be discernable.

A naïve analysis, without principal stratification, of the effect of PEG treatment on BMI measured at 1 year post-baseline is provided in Table 7. For comparability to SACE and to ensure the measurement of the outcome, only those individuals with survival greater than one year post-baseline are considered in this analysis. Parameter estimates and 95% confidence intervals from a linear regression model are presented. These results indicate that without use of the principal stratification framework, we are unable to identify a significant effect of treatment in this dataset. Both the dichotomous treatment model and the time of treatment model return non-significant estimates of treatment effect, regardless of the use of propensity scores. These results provide further evidence in support of the use of principal stratification framework in the presence of censoring by death.

## Discussion

Data from observational registries may be rich in information, but often present incredibly challenging combinations of pre- and post-treatment bias. The proposed causal framework addresses selection bias or confounding due to non-randomized treatment while simultaneously tackling censoring by death. The proposed methodology is inspired by existing problems in the field of observational research and is illustrated with a real-world data example. Using the proposed causal framework, we are able to identify a postive effect of palliative PEG treatment in the ALS clinic registry data. This is particularly meaningful as many other studies that have tried to estimate an effect of this treatment have failed to find a clinical benefit, likely in large part due to the inherent biases and difficulties of data from a fatal disease such as ALS. These results seem to provide a promising role for PEG treatment in the management of ALS, even using sparse data from a clinic registry.

The use of propensity scores or GPS within the principal stratification framework allows for the estimation of an unbiased principal effect of treatment, particularly for observational data or randomized data in which the assumption of no unmeasured confounding is suspect. The removal of bias by inclusion of propensity scores in the principal strata and outcome models is evidenced by the results of the simulation studies presented in Appendix I. It is noteworthy that in order for the principal effect estimate to be unbiased, the assumption of strongly ignorable treatment assignment must hold. In other words, there must be no unmeasured confounders.

Though the effect of the treatment is not significant in the dichotomous treatment model for PEG treatment, a positive and significant effect of treatment is identified in the time of treatment model. As mentioned in earlier sections, it is possible that we are unable to capture the effects of treatment administered at time points further away from the time of outcome measurement due to a waning effect of treatment. A reduction of effect size could suggest that there is an initial effect of PEG, but that as patient health deteriorates over time, the palliative effect of the intervention also decreases. Identifying the treatment effect as it changes over time may be of use to the clinical community, requires more sophisticated techniques, and thus is left for future research.

Overall, the results presented from the application to the ALS data are not sensitive to the assumption of monotonicity. In the data application, this may be due to the small proportion of individuals in *DL* strata when all four strata are considered. When monotonicity is not assumed, most patients are in the *LL* and *DD* strata, with a *LD* and *DL* strata each comprising less than 5% of individuals each. It is conceivable then that reallocating such a small proportion of individuals when removing the *DL* stratum would likely not substantially change the effect estimates of the other strata.

Propensity scores are included as linear predictors of the outcome and principal strata models, which can be considered restrictive when controlling for selection bias and confounding. However, due to the complexity of fitting two models in this methodology, using propensity scores as linear predictors offeres a straight-forward method of controlling for confounding. Adding higher order terms does add some flexibility in the relationships between propensity score and the dependent variables of each of the models as it acts as a polynomial basis regression splines, but other basis functions may be considered in the future for the most effective control for bias. Future research could be devoted to developing methodology that allows for matching or stratification by propensity score or GPS while also using a principal stratification framework.

In the current data analysis from the Emory ALS Clinic, one important confounder that is not available is the Revised ALS Functional Rating Scale (ALSFRS-R) score, a validated instrument that measures the progression of ALS. However, even without the ALSFRS-R score, balance of the observed covariates in Table 3 and the change of effect estimates that occurs when the propensity score is included in the model lend support to the validity in our propensity score methods. Additionally, a sensitivity analysis could be desiged to estimate the bias caused by unmeasured confounders; however the development of such a sensitivity analysis for the proposed methodology is beyond the scope of this paper and could be considered as a direction for future research.

Future consideration may also be given to jointly modeling the propensity score with the outcome model and principal strata model in the Bayesian framework. This would allow the quantities observed by sampling outcome and principal strata models to affect the posterior of propensity score in each MCMC iteration. While this could provide a more robust propensity score adjustment, Zigler *et al.*^15^ show that the feedback between model stages in joint modeling can cause biased causal effect estimates if individual covariates are not also adjusted for in the outcome model^15^. This bias should be accounted for if joint modeling of the three models of outcome, propensity scores, and principal strata is proposed.

## Additional Information

**How to cite this article**: Mishra-Kalyani, P. S. *et al.* Estimating the palliative effect of percutaneous endoscopic gastrostomy in an observational registry using principal stratification and generalized propensity scores. *Sci. Rep.* **6**, 33431; doi: 10.1038/srep33431 (2016).

## Supplementary Material

Supplementary Information

## Figures and Tables

**Table 1:** Individual observed likelihood by observed treatment and survival group.

Observed Group	*Z*_*i*_	*S*_*i*_	*G*_*i*_			
			*LL*	*LD*	*DL*	*DD*
O(1, 1)	1	1	*π*_*LL*,*i*_*f*_*LL*,*i*_	*π*_*LD*,*i*_*f*_*LD*,*i*_	—	—
O(1, 0)	1	0	—	—	*π*_*DL*,*i*_*f*_*DL*,*i*_	*π*_*DD*,*i*_*f*_*DD*,*i*_
O(0, 1)	0	1	*π*_*LL*,*i*_*f*_*LL*,*i*_	—	*π*_*DL*,*i*_*f*_*DL*,*i*_	—
O(0, 0)	0	0	—	*π*_*LD*,*i*_*f*_*LD*,*i*_	—	*π*_*DD*,*i*_*f*_*DD*,*i*_

**Table 2:** Comparison of PEG treated and untreated populations 1 year post-baseline (N = 580).

	Treated (N = 200)		Untreated (N = 380)		
	Mean/P	SD or n	Mean/P	SD or n	p-value
BMI at t^o^	23.87	5.52	24.89	5.63	0.05
Baseline (BL) BMI	24.92	5.89	25.62	5.68	0.19
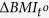	−1.13	2.12	−0.65	2.92	0.04
FVC at t^o^	46.84	21.44	63.49	25.11	<0.01
Baseline (BL) FVC	65.24	25.58	73.98	25.84	<0.01
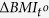	−18.86	22.09	−10.42	16.96	<0.01
Age at Diagnosis	64.91	10.15	61.91	12.17	<0.01
ΔT_DX_ > 30 days	0.21	41	0.19	71	0.68
Proportion Surviving	0.42	83	0.54	206	<0.01
Prop. of Females	0.52	103	0.41	156	0.02
Prop. of Spinal Onset	0.43	86	0.80	304	<0.01

**Table 3:** Balance of Covariates Among Treatment Groups: P-values of Treatment Indicator Effect on Patient Characteristics after Inclusion of Propensity Scores (N = 491).

	Without PS	Standard PS	Generalized PS
Baseline BMI	0.186	0.499	0.185
Baseline FVC	<0.001	0.807	0.002
Age at Diagnosis	0.003	0.457	0.278
Number of Visits	<0.001	0.188	0.007
Spinal Site of Onset	<0.001	0.587	0.113
Female Sex	0.016	0.131	0.717

**Table 4:** SACE of PEG treatment (with 95% credible intervals)on BMI measured 1 year post-baseline (N = 491).

	No Propensity Score		Linear PS or GPS Model	
	Mean	95% CI	Mean	95% CI
*Outcome model including binary treatment indicator only*				
Peg Treatment	−1.21	(−2.51, 0.07)	−0.17	(−1.56, 1.22)
*Outcome model including both binary indicator and time of treatment*				
Time from Treatment to t*	−0.49	(−0.77, −0.21)	−0.34	(−0.60, −0.06)
Peg Treatment	1.84	(−0.18, 3.85)	2.69	(0.82, 4.53)

**Table 5:** Comparison of SACE estimates of PEG treatment (with 95% credible intervals) with and without the monotonicity assumption in the dichotomous treatment model (N = 491).

	PEG Treatment	Effect Estimate
	Monotonicity	All Four Strata
No Propensity Score	−1.37 (−*2*.*69*, −*0*.*02*)	−1.21 (−*2*.*51*, *0*.*07*)
Linear Propensity Score Term	0.40 (−*0*.*93*, *1*.*73*)	−0.17 (−*1*.*56*, *1*.*22*)
Quadratic Propensity Score Terms	0.37 (−*0*.*99*, *1*.*70*)	−0.15 (−*1*.*57*, *1*.*27*)
Cubic Propensity Score Terms	−0.24 (−*1*.*66*, *1*.*20*)	−0.16 (−*1*.*59*, *1*.*25*)

**Table 6:** Comparison of SACE estimates of PEG treatment (with 95% credible intervals) with and without the monotonicity assumption in the time of treatment model (N = 491).

	PEG Treatment Indicator		Time from Treatment to t*	
	Monotonicity	All Strata	Monotonicity	All Strata
No GPS	1.48 (−*0*.*71*, *3*.*57*)	1.84 (−*0*.*18*, *3*.*85*)	−0.50 (−*0*.*19*, −*0*.*80*)	−0.49 (−*0*.*21*, −*0*.*77*)
Linear GPS Term	2.25 (*0*.*19*, *4*.*30*)	2.69 (*0*.*82*, *4*.*53*)	−0.35 (−*0*.*05*, −*0*.*65*)	−0.34 (−*0*.*06*, −*0*.*60*)
Quadratic GPS Terms	2.34 (*0*.*24*, *4*.*40*)	2.88 (*1*.*04*, *4*.*78*)	−0.37 (−*0*.*06*, −*0*.*68*)	−0.38 (−*0*.*10*, −*0*.*66*)
Cubic GPS Terms	2.39 (*2*.*39*, *0*.*28*)	2.40 (*2*.*39*, *0*.*37*)	−0.38 (−*0*.*38*, −*0*.*06*)	−0.38 (−*0*.*38*, −*0*.*07*)

**Table 7:** Estimate of PEG treatment effect (with 95% credible intervals) among survivors on BMI measured at 1 year post-baseline without principal stratification (N = 278).

	No Propensity Score		Linear PS or GPS	
	Mean	95% CI	Mean	95% CI
*Model including binary treatment indicator only*				
Peg Treatment	−1.38	(−2.62, −0.13)	−0.10	(−1.46, 1.25)
*Model including both binary indicator and time of treatment*				
Time from Treatment to t*	−0.01	(−0.01, 0.00)	0.00	(−0.01, 0.00)
Peg Treatment	−0.07	(−2.28, 2.15)	0.75	(−1.42, 2.92)
