# Supplementary material for: Estimating the palliative effect of percutaneous endoscopic gastrostomy in an observational registry using principal stratification and generalized propensity scores
Source: Sci Rep. 2016 Sep 19;6:33431. doi: 10.1038/srep33431 (PMC5027570; doi:10.1038/srep33431)
Supplement: Supplementary Information [file srep33431-s1.pdf]

Estimating the palliative effect of percutaneous endoscopic gastrostomy in an observational registry using principal stratification and generalized propensity scores

Pallavi S. Mishra-Kalyani[1], Brent A. Johnson[2], Jonathan D. Glass[3], and Qi Long[4,*]

[1]Food and Drug Administration, Office of Biostatistics, Silver Spring, 20903, USA
[2]University of Rochester, Department of Biostatistics and Computational Biology, Rochester, 14642, USA
[3]Emory University, Department of Neurology, Atlanta, 30322, USA
[4]Emory University, Department of Biostatistics and Bioinformatics, Atlanta, 30322, USA
[*]qlong@emory.edu

Appendix

Appendix I: Simulation Studies
Appendix II: Observed Data Likelihood
Appendix III: Prior Distributions of Parameters
Appendix IV: Imputation Probabilities for I-Step

Appendix I: Simulation Studies

We evaluate the proposed methods in Monte Carlo simulations of 500 datasets. Each Monte Carlo dataset contains 500 observations with patient characteristics, principal stratum assignment, treatment information, and outcome data generated as described in the following sections. In all simulation scenarios, principal stratum, treatment assignment, and survival status must be determined prior to outcome generation, as Y_i can only exist for those individuals with $S_i = 1$. Therefore, while Y_i is generated for all observations with $G_i = LL$, it can only be generated for observations with $G_i = LD$ and $Z_i = 1$ or $G_i = DL$ and $Z_i = 0$, and it cannot be generated for observations wtih $G_i = DD$ at all.

In each of the 500 Monte Carlo datasets, four variables are generated to represent patient characteristics $\mathbf{D}$. $\mathbf{D_1}$, $\mathbf{D_2}$, $\mathbf{D_4}$ are generated from Uniform distributions of varying ranges ($Unif[1,1]$, $Unif[-2,2]$, and $Unif[0,1]$ respectively). $\mathbf{D_3}$ is generated for each individual using a Bernoulli distribution with $p = 0.5$. Overlapping subsets of these covariates are used in the models that generate principal strata assignment ($\mathbf{D_2}$, $\mathbf{D_3}$, $\mathbf{D_4}$) and the treatment assignment ($\mathbf{D_1}$, $\mathbf{D_2}$, $\mathbf{D_3}$) for each observation.

Principal strata assignment, G_i is generated from a discrete distribution with probabilities generated from a multinomial logit model, as presented in equation 1 of the main text. The parameters of this model, α_g, are selected such that LD is the reference group and the average values of the probabilities of principal strata in simulation population have the preferred relationship $\pi_{LL} > \pi_{LD} > \pi_{DL} > \pi_{DD}$. Specifically the parameter values are $\alpha_{LL} = [0.85, 1.0, -0.5, 0.5]$, $\alpha_{DL} = [-0.55, 1.5, -0.5, 0.25]$, and $\alpha_{DD} = [-1.2, -0.8, -0.5, -0.5]$. Treatment assignment, Z_i, is generated from a Bernoulli distribution with probability $p_{Z,i}$. A logistic regression model is used to determine $p_{Z,i}$, with parameters $\beta = [0.1, 1.5, -1.0, -0.5]$, selected to achieve an average probability that is slightly greater than 0.5 for the simulation population.

Knowledge of principal strata and treatment assignments allow for the extrapolation of survival status S_i for each observation. Finally, an outcome measurement is simulated for those observations with a survival status $S_i = 1$ using outcome distributions, $f_g = N(\mathbf{X}_{1,g}\eta_g, \sigma_g^2)$.. The design matrix for the LL stratum, $\mathbf{X}_{LL} = (1, \mathbf{Z}, \mathbf{D}_1, \mathbf{D}_2, \mathbf{D}_3)$, differs from those of the LD and DL by inclusion of the treatment assignment covariate ($\mathbf{X}_{LD} = X_{DL} = (1, \mathbf{D}_1, \mathbf{D}_2, \mathbf{D}_3)$). The outcome model parameters for each stratum are set to $\eta_{LL} = [6, 4, 1.5, 0.8, 0.4]$, $\eta_{LD} = [1, 1, 0.4, 0.2]$, and $\eta_{DL} = [2, 1, 0.4, 0.2]$.

We also consider Monte Carlo simulations in which the monotonicity assumption holds. Three strata are considered, with the he DL stratum removed from the framework. Parameter values α_g for the strata model and η_g for the outcome model remain the same for LL, LD, and DD. It is noteworthy that the assumptions made for the data generation in each scenario are imposed on the statistical analysis of the data. That is, when data are simulated with a monotonicity assumption, it is analyzed in the same manner; when data is simulated without the monotonicity assumption, no monotonicity assumption is imposed on

the statistical analysis.

The estimated effects of treatment for various dichotomous treatment models are presented in Table A1. These results include simulations with and without the monotonicity assumption and the incorporation of higher order propensity scores. The monotonicity assumption reduces the number of principal strata to three by eliminating the *DL* stratum, with all other models, distributions, and true parameters remaining the same as when the monotonicity assumption is not employed. Propensity score is calculated as the probability of treatment using a logistic regression model for the generated Z_i.

The estimates of effect when a propensity score term is not included in the model with and without monotonicity assumption exhibit substantial bias. However, the inclusion of propensity scores as linear predictors, either singularly or with higher order terms, reduces the bias of the effect estimates considerably. The relative bias of the effect estimate of treatment is approximately 33% in both scenarios with and without the monotonicity assumption when propensity scores are absent, but this relative bias shrinks to 1.9%-3.8% when propensity score terms are present. Additionally, the coverage probabilities in the presence of propensity scores range from 92%-95% in simulations without the monotonicity assumption and 95%-97% in simulations with the monotonicity assumption. While relative bias is smallest and coverage probability is highest when cubic propensity score terms (and corresponding lower order terms) are included in the simulation framework, those models with linear or quadratic propensity score terms also perform well.

Appendix II: Observed Data Likelihood

$$
\begin{aligned}
P(Y|S,Z,G,D,PS) \propto \prod_{i\in O(1,1)} & \left\{ \frac{e^{\mathbf{X}_{2,i}\alpha_{LL}} \sigma_{LL}^{-1} e^{\frac{\left(Y_i - \mathbf{X}_{1,LL,i}\eta_{LL}\right)^2}{2\sigma_{LL}^2}} + \sigma_{LD}^{-1} e^{\frac{\left(Y_i - \mathbf{X}_{1,LD,i}\eta_{LD}\right)^2}{2\sigma_{LD}^2}}}{1 + e^{\mathbf{X}_{2,i}\alpha_{LL}} + e^{\mathbf{X}_{2,i}\alpha_{DL}} + e^{\mathbf{X}_{2,i}\alpha_{DD}}} \right\} \\
\times \prod_{i\in O(0,1)} & \left\{ \frac{e^{\mathbf{X}_{2,i}\alpha_{LL}} \sigma_{LL}^{-1} e^{\frac{\left(Y_i - \mathbf{X}_{1,LL,i}\eta_{LL}\right)^2}{2\sigma_{LL}^2}} + e^{\mathbf{X}_{2i}\alpha_{DL}} \sigma_{DL}^{-1} e^{\frac{\left(Y_i - \mathbf{X}_{1,DL,i}\eta_{DL}\right)^2}{2\sigma_{DL}^2}}}{1 + e^{\mathbf{X}_{2,i}\alpha_{LL}} + e^{\mathbf{X}_{2,i}\alpha_{DL}} + e^{\mathbf{X}_{2,i}\alpha_{DD}}} \right\} \\
\times \prod_{i\in O(1,0)} & \left\{ \frac{e^{\mathbf{X}_{2,i}\alpha_{DL}} + e^{\mathbf{X}_{2,i}\alpha_{DD}}}{1 + e^{\mathbf{X}_{2,i}\alpha_{LL}} + e^{\mathbf{X}_{2,i}\alpha_{DL}} + e^{\mathbf{X}_{2,i}\alpha_{DD}}} \right\} \\
\times \prod_{i\in O(0,0)} & \left\{ \frac{1 + \mathbf{X}_{2,i} e^{\alpha_{DD}}}{1 + e^{\mathbf{X}_{2,i}\alpha_{LL}} + e^{\mathbf{X}_{2,i}\alpha_{DL}} + e^{\mathbf{X}_{2,i}\alpha_{DD}}} \right\}
\end{aligned}
$$

Appendix III: Prior Distributions of Parameters

1. $p(\alpha_g) \propto 1$

Table A1. Results of simulations with binary treatment indicator

Treatment Effect Estimate (LL Stratum)	Relative Bias (%)	Mean SE	Empirical SD	Coverage Probability
Without Monotonicity (All 4 Principal Strata)				
No PS Term	33.63	0.266	0.265	0.00
Linear PS Term	3.78	0.224	0.221	0.93
Quadratic PS Terms	2.17	0.221	0.219	0.92
Cubic PS Terms	1.93	0.225	0.221	0.95
With Monotonicity Assumption (No DL Stratum)				
No PS Term	32.82	0.190	0.190	0.00
Linear PS Term	2.49	0.170	0.170	0.95
Quadratic	1.88	0.171	0.171	0.97
Cubic	2.03	0.172	0.172	0.96

2. $\eta_g \sim Normal_p\left(\mu_g, \sigma_g^2 \mathbf{V}_g\right)$

$p\left(\eta_g\right) \propto |\sigma_g^2 V_g|^{-\frac{1}{2}} e^{-\frac{1}{2\sigma_g^2}\left(\eta_g-\mu_g\right)^T \mathbf{V}_g^{-1}\left(\eta_g-\mu_g\right)}$

3. $\sigma_g^2 \sim InverseGamma\left(\nu_g, \omega_g\right)$

$p\left(\sigma_g^2\right) \propto \sigma_g^{2(-\nu_{LL}-1)} exp\left(-\frac{\omega_{LL}}{\sigma_{LL}^2}\right)$

Appendix IV: Imputation Probabilities for I-Step

1. For $O(1,1)$:

$$P\left(G_i^{(k+1)} = LL\right) = \rho_{11,i}^{(k+1)} = \frac{\pi_{LL,i}^{(k)} f_{LL,i}^{(k)}}{\pi_{LL,i}^{(k)} f_{LL,i}^{(k)} + \pi_{LD,i}^{(k)} f_{LD,i}^{(k)}}$$

$$= \frac{\frac{e^{\mathbf{X}_{2,i}\alpha_{LL}}}{\sigma_{LL}^{(k)}} \exp\left(\frac{\left(Y_i - \mathbf{X}_{1,LL,i}\eta_{LL}\right)^2}{2\sigma_{LL}^2}\right)}{\frac{e^{\mathbf{X}_{2,i}\alpha_{LL}}}{\sigma_{LL}^{(k)}} \exp\left(\frac{\left(Y_i - \mathbf{X}_{1,LL,i}\eta_{LL}\right)^2}{2\sigma_{LL}^2}\right) + \frac{e^{\mathbf{X}_{2,i}\alpha_{LD}}}{\sigma_{LD}^{(k)}} \exp\left(\frac{\left(Y_i - \mathbf{X}_{1,LD,i}\eta_{LD}\right)^2}{2\sigma_{LD}^2}\right)}$$

$$P\left(G_i^{(k+1)} = LD\right) = 1 - \rho_{11,i}^{(k+1)}$$

2. For $O(0,1)$:

$$P\left(G_i^{(k+1)} = LL\right) = \rho_{01,i}^{(k+1)} = \frac{\pi_{LL,i}^{(k)} f_{LL,i}^{(k)}}{\pi_{LL,i}^{(k)} f_{LL,i}^{(k)} + \pi_{DL,i}^{(k)} f_{DL,i}^{(k)}}$$

$$= \frac{\frac{e^{\mathbf{X}_{2,i}\alpha_{LL}}}{\sigma_{LL}^{(k)}} \exp\left(\frac{\left(Y_i - \mathbf{X}_{1,LL,i}\eta_{LL}\right)^2}{2\sigma_{LL}^2}\right)}{\frac{e^{\mathbf{X}_{2,i}\alpha_{LL}}}{\sigma_{LL}^{(k)}} \exp\left(\frac{\left(Y_i - \mathbf{X}_{1,LL,i}\eta_{LL}\right)^2}{2\sigma_{LL}^2}\right) + \frac{e^{\mathbf{X}_{2,i}\alpha_{DL}}}{\sigma_{DL}^{(k)}} \exp\left(\frac{\left(Y_i - \mathbf{X}_{1,DL,i}\eta_{DL}\right)^2}{2\sigma_{DL}^2}\right)}$$

$$P\left(G_i^{(k+1)} = DL\right) = 1 - \rho_{01,i}^{(k+1)}$$

3. For $O(1,0)$:

$$P\left(G_i^{(k+1)} = DD\right) = \rho_{10,i}^{(k+1)} = \frac{\pi_{DD,i}^{(k)}}{\pi_{DD,i}^{(k)} + \pi_{DL,i}^{(k)}}$$

$$= \frac{e^{\mathbf{X}_{2,i}\alpha_{DD}}}{e^{\mathbf{X}_{2,i}\alpha_{DD}} + e^{\mathbf{X}_{2,i}\alpha_{DL}}}$$

$$P\left(G_i^{(k+1)} = DL\right) = 1 - \rho_{10,i}^{(k+1)}$$

4. For $O(0,0)$:

$$P\left(G_i^{(k+1)} = DD\right) = \rho_{00,i}^{(k+1)} = \frac{\pi_{DD,i}^{(k)}}{\pi_{DD,i}^{(k)} + \pi_{LD,i}^{(k)}}$$

$$= \frac{e^{\mathbf{X}_{2,i}\alpha_{DD}}}{e^{\mathbf{X}_{2,i}\alpha_{DD}} + e^{\mathbf{X}_{2,i}\alpha_{LD}}}$$

$$P\left(G_i^{(k+1)} = LD\right) = 1 - \rho_{00,i}^{(k+1)}$$
